# Kinetics of immune responses to the AZD1222/Covishield vaccine with varying dose intervals in Sri Lankan individuals

**DOI:** 10.1002/iid3.592

**Published:** 2022-03-22

**Authors:** Chandima Jeewandara, Inoka Sepali Aberathna, Laksiri Gomes, Pradeep Darshana Pushpakumara, Saubhagya Danasekara, Dinuka Guruge, Thushali Ranasinghe, Banuri Gunasekera, Achala Kamaladasa, Heshan Kuruppu, Gayasha Somathilake, Osanda Dissanayake, Nayanathara Gamalath, Dinithi Ekanayake, Jeewantha Jayamali, Deshni Jayathilaka, Anushika Mudunkotuwa, Michael Harvie, Thashmi Nimasha, Ruwan Wijayamuni, Lisa Schimanski, Pramila Rijal, Tiong K. Tan, Tao Dong, Alain Townsend, Graham S. Ogg, Gathsaurie Neelika Malavige

**Affiliations:** ^1^ Allergy Immunology and Cell Biology Unit, Department of Immunology and Molecular Medicine University of Sri Jayewardenepura Nugegoda Sri Lanka; ^2^ Colombo Municipal Council Colombo Sri Lanka; ^3^ MRC Human Immunology Unit, MRC Weatherall Institute of Molecular Medicine, University of Oxford Oxford UK; ^4^ Centre for Translational Immunology, Chinese Academy of Medical Sciences Oxford Institute, University of Oxford Oxford UK

**Keywords:** antibodies, AZD1222, COVID‐19, dosing intervals, immunogenecity, T cells, vaccines, variants

## Abstract

**Background:**

To understand the kinetics of immune responses with different dosing gaps of the AZD1222 vaccine, we compared antibody and T cell responses in two cohorts with two different dosing gaps.

**Methods:**

Antibodies to the SARS‐CoV‐2 virus were assessed in 297 individuals with a dosing gap of 12 weeks, sampled 12 weeks post second dose (cohort 1) and in 77 individuals with a median dosing gap of 21.4 weeks (cohort 2) sampled 6 weeks post second dose. ACE2‐blocking antibodies (ACE2‐blocking Abs), antibodies to the receptor‐binding domain (RBD) of  variants of concern (VOC), and ex vivo T cell responses were assessed in a subcohort.

**Results:**

All individuals (100%) had SARS‐CoV‐2‐specific total antibodies and 94.2% of cohort 1 and 97.1% of cohort 2 had ACE2‐blocking Abs. There was no difference in antibody titers or positivity rates in different age groups in both cohorts. The ACE2‐blocking Abs (*p* < .0001) and antibodies to the RBD of the VOCs were significantly higher in cohort 2 compared to cohort 1. 41.2% to 65.8% of different age groups gave a positive response by the hemagglutination assay to the RBD of the ancestral virus and VOCs in cohort 1, while 53.6%–90% gave a positive response in cohort 2. 17/57 (29.8%) of cohort 1 and 17/29 (58.6%) of cohort 2 had ex vivo interferon (IFN)γ ELISpot responses above the positive threshold. The ACE2‐blocking antibodies (Spearman's *r* = .46, *p* = .008) and ex vivo IFNγ responses (Spearman's *r* = .71, *p* < .0001) at 12 weeks post first dose, significantly correlated with levels 12 weeks post second dose.

**Conclusions:**

Both dosing schedules resulted in high antibody and T cell responses post vaccination, although those with a longer dosing gap had a higher magnitude of responses, possibly as immune responses were measured 6 weeks post second dose compared to 12 weeks post second dose.

## Graphical abstract

We found that those who had a wider dosing gap (20 weeks compared to 12 weeks) had higher antibody and T cell responses, 6 months post first dose of the AZD1222 vaccine, possibly as immune responses were measured 6 weeks post second dose compared to 12 weeks post second dose.

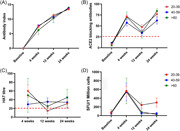

## 1 BACKGROUND

The ChAdOx1 nCoV‐19 (AZD1222) vaccine, is a nonreplicating chimpanzee adenovirus vector vaccine, containing the sequence for the spike protein of the ancestral SARS‐CoV‐2 virus.^1^ Although the vaccine was initially administered with a dosing gap of 4 weeks between the two doses, subsequently, the dosing gap was increased to 12 weeks, as it was shown to increase the efficacy of the vaccine.^2^ However, many countries increased the dosing gap to 16 weeks to administer a single dose to a larger population^3^, ^4^ and also due to the delay in obtaining adequate vaccines for administering the second dose on time.^5^ It was later shown that an increase in the gap between the two doses beyond 12 weeks, and even up to 45 weeks, resulted in higher antibody titers after the second dose of the vaccine.^6^

AZD1222 was the first vaccine to be rolled out in Sri Lanka, with the immunization of the health care workers. However, after it was rolled out to the public, due to the delay in obtaining the second dose, most individuals received their second dose 20 weeks after obtaining their first dose. We showed that at ≥16 weeks postimmunization with a single dose of AZD1222, 93.7% of those >70 years had SARS‐CoV‐2‐specific antibodies, although ACE2 receptor‐blocking antibodies (those that correlate with neutralizing antibodies) was significantly less in those >60 years of age.^5^ However, robust memory T cell and B cell responses were seen in over 90% of individuals. Although it has been shown that an increase in the gap between the two doses subsequently led to higher antibody titers,^6^ there are limited data in the differences in dosing gaps on antibody responses to SARS‐CoV‐2 variants of concern (VOCs), differences in antibody responses in different age groups as well as with those with comorbidities, and also the influence of the dosing gap on T cell responses.

Currently, many studies have shown that there is waning of immunity with many of the COVID‐19 vaccines following the second dose.^7^, ^8^, ^9^ Due to an increased rate of breakthrough infections, some of which led to hospitalization and death, due to waning of immunity, a booster dose is recommended to elderly and immunocompromised individuals by many authorities.^10^, ^11^ While waning of antibody levels alone does not necessarily imply waning of efficacy^12^ and protection, it is important to find out if different dosing schedules lead to differences in the quality and quantity of immune responses and thus, an impact on the duration of immunity. To answer these questions, we compared the immune responses of two cohorts of Sri Lankan individuals who received the AZD1222 vaccine at 12‐week dosing intervals and another cohort who received the vaccine at a median of 21.1 weeks dosing interval. We also investigated the kinetics of antibody and T cell responses in the first cohort (12‐week dosing interval),^13^ who consisted of health care workers, to find out if there was waning of immunity.

## 2 METHODS

Three hundred and thirty‐six health care workers (HCWs) (cohort 1) who received their first dose of AZD1222/Covishield vaccine between January 29 to  February 5, 2021 and their second dose between  April 23 to May 5 were included in the study (12 weeks interval between the two doses) 3 months after receiving the second dose of the vaccine (6 months after the first dose). To compare the influence of the gap between the two doses on the immunogenicity of the vaccine, another cohort of individuals (*n* = 88) in the community (cohort 2), who received their first dose between February 15 to March 4, 2021 and the second dose between June 1 to August 4, 2021 were included in the study 6 weeks after their second dose (17–24 weeks interval between the two doses). Enrolling both cohorts 12 weeks post second dose was difficult due to the planned rollout of booster doses in Sri Lanka since October 2021.

Demographics and the presence of comorbidities, such as diabetes, hypertension, cardiovascular disease, and chronic kidney disease were determined by a self‐administered questionnaire at the time of recruitment from all participants.

Ethics approval was obtained from the Ethics Review Committee of the University of Sri Jayewardenepura.

### 2.1 Detection of SARS‐CoV‐2‐ specific total antibodies

The presence of SARS‐COV‐2‐specific total antibodies (IgM, IgA, and IgG) were detected by using the Wantai SARS‐CoV‐2 Ab ELISA (Beijing Wantai Biological Pharmacy Enterprise), which detects antibodies to the receptor‐binding domain (RBD) of the spike protein. The assay was carried out according to the manufacturer's instructions and the antibody index (an indirect measure of the antibody titer) was calculated by dividing the absorbance of each sample by the cutoff value, according to the manufacturer's instructions.

### 2.2 Surrogate virus neutralization test to detect ACE2‐blocking antibodies (ACE2‐blocking Abs)

The surrogate virus neutralization test (sVNT), which has been shown to correlate with the presence of neutralizing antibodies was used to measure ACE2R‐Abs as previously described according to the manufacturer's instructions (Genscript Biotech).^14^ This measures the percentage of inhibition of binding of the RBD to recombinant ACE2 and an inhibition percentage ≥25% in a sample was considered as positive for ACE2‐blocking Abs.^15^

### 2.3 Hemagglutination test (HAT) to detect antibodies to the RBD

The HAT was carried out as previously described using the B.1.1.7 (N501Y), B.1.351 (N501Y, E484K, K417N), and B.1.617.2 versions of the IH4‐RBD reagents,^16^ which included the relevant amino acid changes introduced by site‐directed mutagenesis. The assays were carried out and interpreted as previously described and a titer of 1:20 was considered as a positive response.^13^, ^17^ The HAT titration was performed using seven doubling dilutions of serum from 1:20 to 1:1280, to determine the presence of RBD‐specific antibodies. The RBD‐specific antibody titer for the serum sample was defined by the last well in which the complete absence of “teardrop” formation was observed. A titer of 1:20 was considered as a positive response, as previously determined.^17^

### 2.4 Assays to determine antibodies to the N protein

Qualitative detection of antibodies to SARS‐CoV‐2 nucleocapsid (N) antigen in human serum was performed using Elecsys® Anti‐SARS‐CoV‐2 electrochemiluminescence immunoassay (Cat: 09 203 095 190, Roche Diagnostics) in Cobas e 411 analyzer (Roche Diagnostics). A cutoff index (COI) ≥ 1.0 was interpreted as reactive and COI < 1.00 was considered nonreactive as per the kit guidelines.

### 2.5 Ex vivo interferon (IFN)γ ELISpot assays

Ex vivo IFNγ ELISpot assays were carried out using freshly isolated peripheral blood mononuclear cells (PBMC) obtained from 57 individuals. Individuals for T cell assays were recruited from those in whom these assays had been carried out at 1 month following the first dose of the vaccine.^18^ Two pools of overlapping peptides named S1 (peptide 1–130) and S2 (peptide 131–253) covering the whole spike protein (253 overlapping peptides) were added at a final concentration of 10 µM and incubated overnight as previously described.^19^, ^20^ All peptide sequences were derived from the wild‐type consensus and were tested in duplicate. 100,000 cells/well were added per well. Phytohemagglutinin was included as a positive control and media alone was used as a negative control (Figure S1). Briefly, ELISpot plates (Millipore Corp.) were coated with anti‐human IFNγ antibody overnight (Mabtech AB). The plates were incubated overnight at 37°C and 5% CO_2_. The cells were removed, and the plates developed with a second biotinylated antibody to human IFNγ and washed a further six times. The plates were developed with streptavidin‐alkaline phosphatase (Mabtech AB) and colorimetric substrate, the spots were enumerated using an automated ELISpot reader (AID Germany). Background (PBMCs plus media alone) was subtracted and data were expressed as the number of spot‐forming units (SFU) per 10^6^ PBMCs. A positive response was defined as mean ± 2 SD of the background responses.

### 2.6 Statistical analysis

The 95% confidence intervals for seropositivity for each age category were calculated using the R software (version 4.0.3) and R‐studio (version 1.4.1106). Pearson *χ* ^2^ Association tests were performed at a confidence level of 95% using the R software to identify the statistically significant associations of the age categories and the sex of the individuals with seropositivity. Kruskal–Wallis test was used to determine the differences between the levels of antibodies between different age groups. Spearman's correlation coefficient was used to determine the correlation between antibody, T cell responses, and the age of an individual.

## 3 RESULTS

### 3.1 SARS‐CoV‐2 total antibody responses in individuals with different dosing intervals

**Figure 1:**
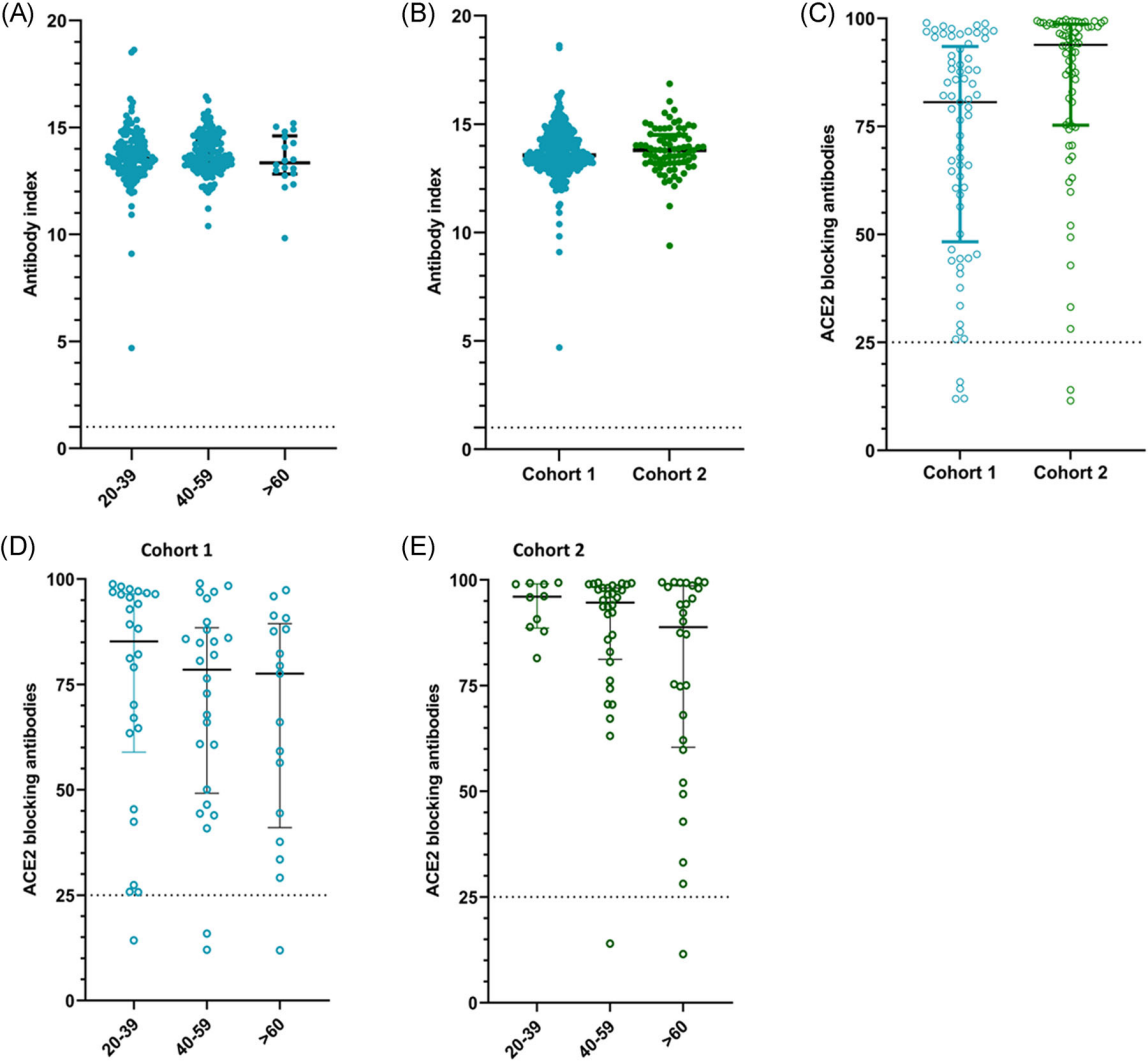
SARS‐CoV‐2‐specific antibodies in individuals of different age groups in cohort 1 and cohort 2. SARS‐CoV‐2‐specific total antibodies were measured in 20–39 years old (*n* = 129), 40–59 years old (*n* = 152), and those >60 years of age (*n* = 16) by ELISA in cohort 1, and no significant difference was seen between the age groups (*p* = .06) based on the Kruskal–Wallis test (A). SARS‐CoV‐2‐specific total antibodies were measured in cohort 1 (*n* = 297) and cohort 2 (*n* = 77) and no significant difference was seen based on the Mann–Whitney test (B). The ACE2 receptor‐blocking antibodies were measured by the surrogate virus neutralizing test in cohort 1 (*n* = 69) and cohort 2 (*n* = 70), which was significantly different (*p* < .0001) (C). The ACE2 receptor‐blocking antibodies were measured in cohort 1 in 20–39 years old (*n* = 26), 40–59 years old (*n* = 26), and >60 years old (*n* = 17) and no significant difference was seen (*p* = .41) between the age groups based on the Kruskal–Wallis test (D). The ACE2 receptor‐blocking antibodies were also in cohort 2 in 20–39 years old (*n* = 10), 40–59 years old (*n* = 32), and >60 years old (*n* = 28) and no significant difference was seen (*p* = .30) between the age groups based on the Kruskal–Wallis test (E). All tests were two‐tailed. The lines indicate the median and the interquartile range. All data points of cohort 1 are shown in blue and in cohort 2 in green

At 3 months since receiving the second dose (6 months after the first dose), all of the 336 (100%) of the HCWs (cohort 1) had SARS‐CoV‐2‐specific total antibodies (IgM, IgG, and IgA). Antibodies to the N protein were tested in both cohorts (cohort 1 and cohort 2) to determine if they had been infected during the 6 months since obtaining the first dose and 39/336 (11.6%) were excluded from further analysis due to the presence of antibodies to the N protein. Therefore, further analysis was carried out in those who were not found to have the natural infection during this period (*n* = 297). There was no significant difference (*p* = .79) between the antibody titers in the three different age groups (20–39, 40–59, and >60 years) in this cohort 1 (Figure 1A).

The second cohort was recruited at 6 weeks since receiving the second dose (6–7 months after the first dose) and the mean gap between the two doses in this cohort was 21.14 weeks (SD ± 1.95 weeks). Of this cohort, 11/88 (12.5%) had antibodies to the N protein and were considered as having been infected with the SARS‐CoV‐2 virus. All of the 77 individuals (100%) of this cohort (cohort 2) also had SARS‐CoV‐2‐specific total antibodies. There was no significant difference between the antibody titers in cohort 1 compared to cohort 2 (*p* = .3488) (Figure 1B). As seen with cohort 1, there was no significant difference (*p* = .5716) between the antibody titers in the three different age groups (20–39, 40–59, and >60 years) in cohort 2. In both cohorts, there was no significant difference (*p* = .96–.99) in the seropositivity rates or the antibody levels (indicated by antibody indices) in those with comorbidities compared to those who did not have comorbidities.

### 3.2 SARS‐CoV‐2‐specific ACE2‐blocking Abs in the two cohorts with different dosing intervals

The sVNT that measures ACE2‐blocking Abs was carried out in a subset of individuals of cohort 1 (*n* = 69) and cohort 2 (*n* = 70). In cohort 1 (those with a 12‐week gap between the two doses), 65/69 (94.2%) gave a positive result for the presence of ACE2‐blocking Abs, while 68/70 (97.1%) in cohort 2 gave a positive response. The positivity rates were found to be significantly different, higher in cohort 1 than cohort 2 (Mann–Whitney *U* = 1472, *p* < .0001) and cohort 2 had significantly higher titers (median 93.7, interquartile range [IQR] 75.3%–98.7% of inhibition) compared to cohort 1 (median 80.6, IQR 48.3%–93.5% of inhibition (Figure 1C). The ACE2‐blocking Ab levels in the three different age groups in cohort 1 and cohort 2 are shown in Table 1. There was no significant difference between the ACE2‐blocking Abs in different age groups in cohort 1 (*p* = .41) (Figure 1D) or cohort 2 (*p* = .30) (Figure 1E).

**Table 1:** ACE2R‐Ab positivity rates and the ACE2R‐Ab levels in different age groups in cohort 1 and cohort 2

Age groups	Presence of ACE2R‐blocking Abs	% of inhibition as given by the sVNT assay (median, IQR)
Cohort 1		
20–39 (*n* = 26)	25 (96.1%)	(85.2, 96.4 − 63.7 = 32.7)
40–59 (*n* = 26)	24 (92.3%)	(78.5, 87.5 − 52.7 = 34.8)
>60 (*n* = 17)	16 (94.1%)	(77.6, 88.1 − 44.4 = 43.7)
Cohort 2		
20–39 (*n* = 10)	10 (100%)	(96.0, 99.0 − 89.3 = 9.6)
40–59 (*n* = 32)	31 (96.9%)	(94.6, 98.1 − 82.4 = 15.7)
>60 (*n* = 28)	27 (96.4%)	(88.8, 98.4 − 61.5 = 36.9)

Abbreviations: ACE2R‐Ab, ACE2‐blocking antibodies; IQR, interquartile range.

### 3.3 HAT to detect antibodies to the RBD of SARS‐CoV‐2 and its VOCs

**Figure 2:**
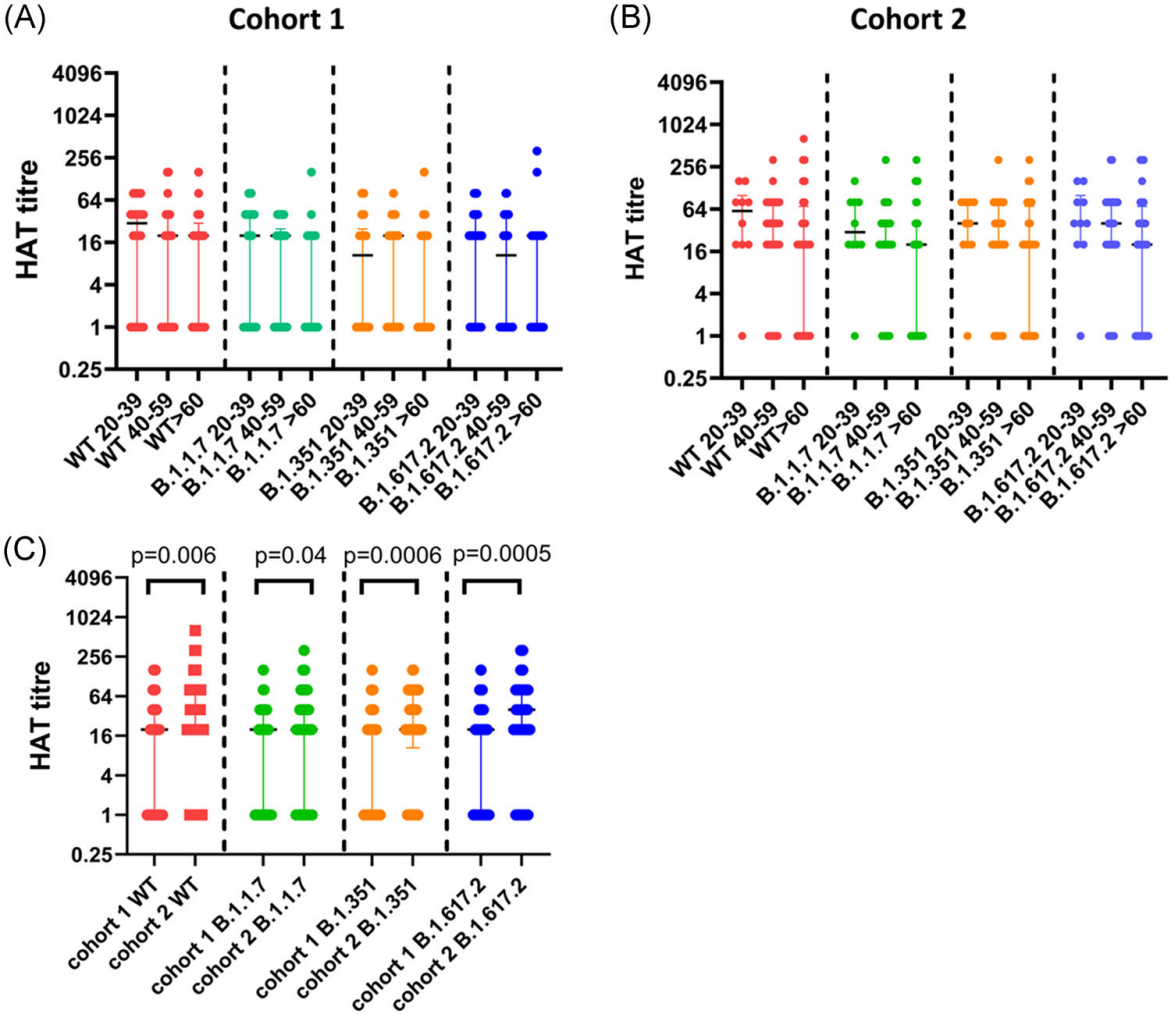
Antibodies to the receptor‐binding domain (RBD) of the ancestral SARS‐CoV‐2 virus (WT) and variants of concern (VOC) in cohorts 1 and 2. Antibodies to the RBD of the WT and VOCs were measured in cohort 1 in 20–39 years old (*n* = 26), 40 to 59 years old (*n* = 26), and >60 years old (*n* = 17) and no significant difference was seen between the different age groups for responses to the RBDs of different VOCs (A). Antibodies to the RBD of the WT and VOCs were also measured in cohort 2 in 20–39 years old (*n* = 10), 40–59 years old (*n* = 32), and >60 years old (*n* = 28) and no significant difference was seen between the different age groups for responses to the RBDs of different VOCs (B). The hemagglutination test (HAT) titers for the WT, B.1.1.7, B.1.351, and B.1.617.2 were compared between the two cohorts (C). Individuals in cohort 2 had significantly higher HAT titers to the WT and all VOCs analyzed by the Mann–Whitney test. All tests were two‐tailed. The lines indicate the median and the interquartile range

The HAT assay was carried out to measure positivity rates and the antibody titers to the ancestral strain (WT), and the VOCs B.1.1.7, B.1.351, and B.1.617.2 in cohort 1 (*n* = 69) and in cohort 2 (*n* = 70). These are the same individuals in whom ACE2 receptor‐blocking antibodies were measured. The proportion of individuals who gave a positive result for the WT and the VOCs is shown in Table 2. As determined by the Friedman test, in cohort 1, the HAT titers for different VOCs were not found to be significantly different in any age group: 20–39 years (*p* = .068), 40–59 years (*p* = .658), and the >60 year age group (*p* = .251) (Figure 2A). The lowest titers were seen for B.1.351, while those who were in the 40–59 age group had low titers for B.1.617.2. In cohort 2, again there was no difference in the HAT titers between WT and VOCs (Figure 2B).

**Table 2:** Positivity rates and titers for WT and SARS‐CoV‐2 VOCs in different age groups in cohort 1 and cohort 2 were measured by the hemagglutination assay

Age groups	WT	B.1.1.7 (alpha)	B.1.351 (beta)	B.1.617.2 (delta)
Cohort 1				
20–39 (*n* = 26)	17 (65.38%)	15 (57.69%)	13 (50%)	17 (65.38%)
40–59 (*n* = 26)	14 (53.85%)	15 (57.69%)	14 (53.85%)	13 (50%)
>60 (*n* = 17)	10 (58.82%)	8 (47.06%)	8 (47.06%)	7 (41.18%)
Cohort 2				
20–39 (*n* = 10)	9 (90%)	9 (90%)	9 (90%)	9 (90%)
40–59 (*n* = 32)	26 (81.25%)	25 (78.13%)	25 (78.13%)	27 (85.29%)
>60 (*n* = 28)	19 (67.86%)	15 (53.57%)	19 (67.86%)	19 (67.86%)

The HAT titers for the WT (*p* = .006), B.1.1.7 (*p* = .04), B.1.351 (*p* = .0006), and B.1.617.2 (*p* = .0005) were significantly higher in those in cohort 1 compared to cohort 2 (Figure 2C).

### 3.4 Ex vivo ELISpot responses in the two cohorts with different dosing schedules

**Figure 3:**
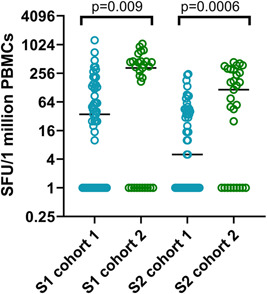
Ex vivo IFNγ ELISpots responses to the overlapping peptides of the spike protein in the two cohorts. Ex vivo interferon (IFN)γ ELISpots responses were measured to the S1 and S2 overlapping pool of peptides in 57 individuals in cohort 1 (blue) and 29 individuals in cohort 2 (green). The frequency of ex vivo IFNγ ELISpot responses were significantly higher for the S1 pool of overlapping peptides (*p* = .009) and the S2 pool of overlapping peptides (*p* = .0006) in cohort 2 compared to cohort 1, based on the Mann–Whitney test. All tests were two‐tailed

To investigate the T cell responses in these two cohorts, we carried out ex vivo IFNγ ELISpot responses in cohort 1 (*n* = 57) and in cohort 2 (*n* = 29). In cohort 1, the positive threshold was set at 110 SFU/1 million PBMCs and accordingly, 17/57 (29.8%) gave a positive response. In cohort 2, the threshold for a positive response was set at 272 SFU/1 million cells. Accordingly, 17/29 (58.6%) gave a positive response. The frequency of ex vivo IFNγ ELISpot responses were significantly higher for the S1 pool of overlapping peptides (*p* = .009) and the S2 pool of overlapping peptides (*p* = .0006) in cohort 2 compared to cohort 1 (Figure 3).

The responses to the S1 pool of overlapping peptides (median 35, IQR 0–132.5 SFU/1 million PBMCs) were significantly higher (*p* < .0001) than for the S2 pool of overlapping peptides (median 5, IQR 0–45 SFU/1 million PBMCs) in cohort 1. In cohort 2, although the responses to the S1 pool was higher (median 330, IQR 0–452.5 SFU/1 million PBMCs) than those for S2 (median 115, IQR 0–305 SFU/1 million PBMCs), this was not significant (*p* = .06).

### 3.5 Kinetics of antibody and T cell responses in cohort 1 over time

**Figure 4:**
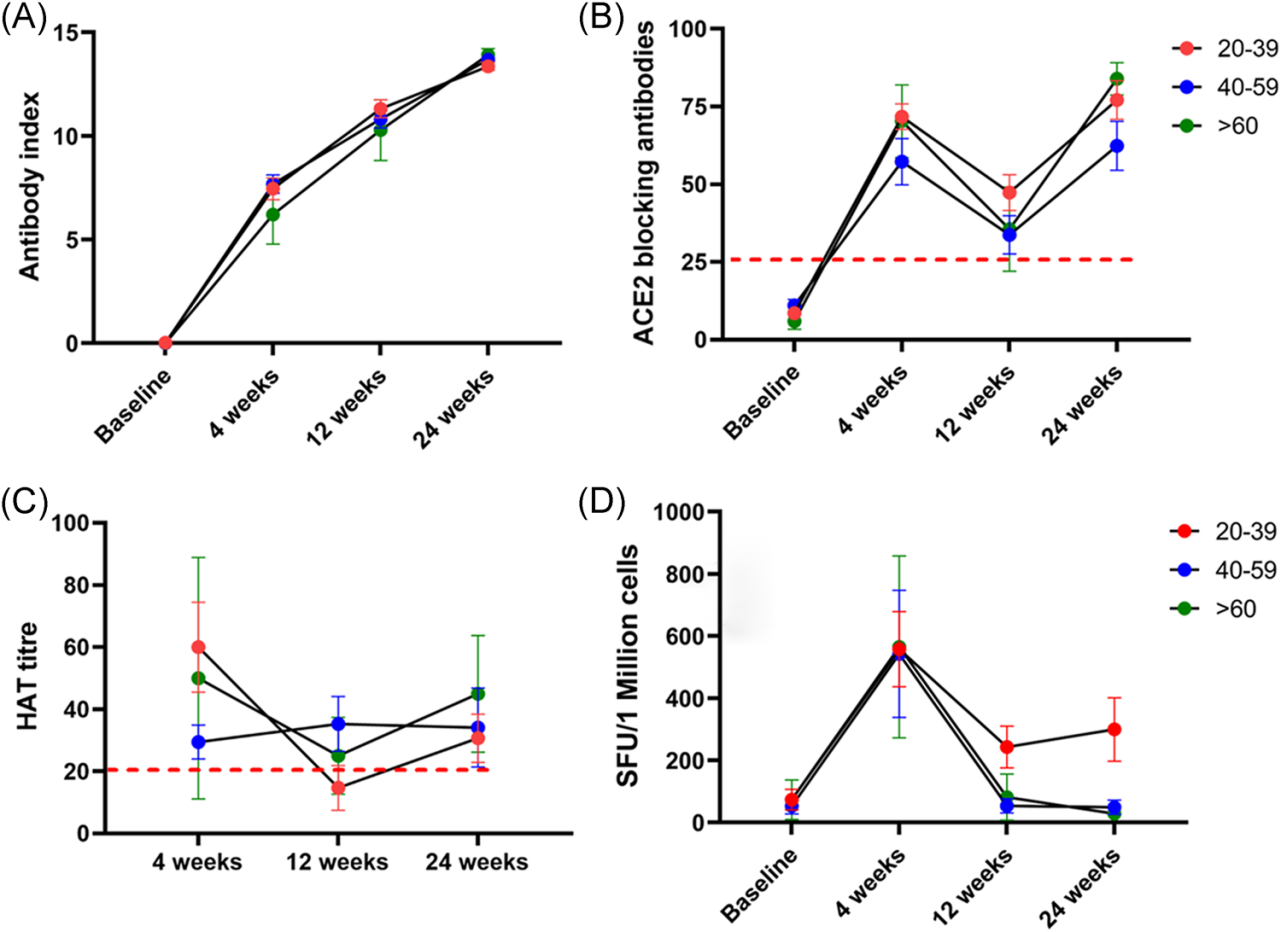
Kinetics of antibody and T cell responses over time in cohort 1 (dosing gap of 12 weeks). SARS‐CoV‐2 total antibodies were measured in 171 individuals (73 in the 29–39 age group, 87 in the 40–59 age group, and 11 in the >60 age group), at baseline, 4 weeks after the first dose, 12 weeks post first dose, and 12 weeks post second dose by ELISA (A). ACE2 receptor antibodies were measured by the surrogate virus neutralizing test in 33 individuals (16 in 29–30 age group, 12 in 40–59, and 5 in >60 age group) (B). Antibodies to the receptor‐binding domain of the WT was measured by the hemagglutination assay test (HAT) in 40 individuals (C). Ex vivo IFNγ ELISpot responses to the S protein overlapping pool of peptides were measured in 36 individuals, with 16 in the 20–39 age group, 17 in the 40–59 age group, and 3 in the >60 years. There was no difference in responses between 6 and 16 weeks (D). The lines indicate the mean and the error bars the standard error of the mean. All tests were two‐tailed. SFU, spot‐forming unit

The kinetics of antibodies and T cell responses could only be studied in cohort 1, as we had data at baseline, 4 weeks after the first dose, 12 weeks after the first dose, and 12 weeks after the second dose (6 months after the first dose) only for cohort 1. Only 171 individuals (N antibody negative) were included in the analysis for kinetics of the SARS‐CoV‐2 total antibodies as all four time points were available only in these individuals. Of the 171 individuals, 73 were in the 29–39 age group, 87 in the 40–59 age group, and 11 in the >60 age group. The SARS‐CoV‐2 total antibodies were significantly higher from the time from obtaining the second dose to 12 weeks later (*p* < .0001) (Figure 4A). However, while the SARS‐CoV‐2‐specific total antibodies rose with time in all age groups, this rise was only significant in the 40–59 age group (*p* = .001), while there was no significant difference in those in the 20–39 age group and >60 age group, in the levels from 12 weeks post first vaccine and 12 weeks post second vaccine (Figure 4A). In the 20–39 age group and the 40–59 age group the antibody levels were significantly higher at 12 weeks post second dose (24 months post first dose) than at 4 weeks post first dose (*p* < .0001). In the >60 age group, although the levels at 12 weeks post second dose was significantly higher than that of 4 weeks post first dose (*p* = .049), the levels were still lower.

The ACE2‐blocking Ab levels were only available in 33 individuals (16 in 29–30 age group, 12 in 40–59, and 5 in >60 age group), in cohort 1 for all four time points. Using the Friedman test we found that the ACE2‐Ab levels were significantly higher (*p* < .0001) at 12 weeks post second dose compared to 12 weeks post first dose (Figure 4B). This rise in ACE2‐blocking Abs was only significantly higher in the 20–39 age group (*p* = .002), but not in the other two age groups. The antibodies to the RBD of the WT was also assessed over time in this cohort (*n* = 40), and there was no significant difference (*p* = .59) in the antibody titers to the RBD of the WT, assessed by the hemagglutination assay from 12 weeks post second dose compared to 12 weeks post first dose (Figure 4C).

The kinetics of ex vivo IFNγ ELISpot responses were assessed in 36 individuals over time (16 in the 20–39 age group, 17 in the 40–59 age group, and 3 in >60 years). While the ex vivo spike protein‐specific (overlapping peptide) responses increased with time, there was no significant difference (*p* > .99) in responses 12 weeks post second dose compared to 12 weeks post first dose (Figure 4D), in any of the three age groups. Statistical analysis was not carried out between individual age groups as there were only three individuals >60 years of age. However, the T cell responses were higher in those in the 20–39 age group.

### 3.6 Association of immune responses to the first dose with those of immune responses following post second dose of the vaccine

In cohort 1, the antibody levels at 12 weeks post single dose significantly correlated with the SARS‐CoV‐2 total antibodies at 12 weeks post second dose (Spearman's *r* = .22, *p* = .001). However, in further analysis, this correlation was only seen in the 20–39 age group (Spearman's *r* = .37, *p* = .0003), whereas no significant correlation was seen in other age groups. However, no such correlation was seen in the total antibodies at ≥16 weeks post first dose and 6 weeks post second dose in cohort 2 (Spearmans *r* = .03, *p* = .82).

In cohort 1 (Spearman's *r* = .46, *p* = .008) the ACE2‐blocking Abs at 12 weeks post first dose, significantly correlated with levels 12 weeks post second dose. A significant correlation was seen in cohort 2 (Spearman's *r* = .41, *p* = .001) as well in antibody levels at ≥16 weeks post first dose and 6 weeks post second dose. The ex vivo IFNγ ELISpot responses showed the strongest correlation in cohort 1 in 12 weeks post first dose and 12 weeks post second dose (Spearman's *r* = .71, *p* < .0001). Data were not available to carry out this analysis for cohort 2.

## 4 DISCUSSION

In this study, we have compared the antibody and T cell responses of individuals with two dosing schedules, 6 months after receiving the first dose of the AZD1222 vaccine. In the first cohort with a dosing gap of 12 weeks, individuals were recruited 12 weeks post second dose, while in cohort 2, with a dosing gap of a median of 21.4 weeks, individuals were recruited 6 weeks post second dose. We found that all individuals in both cohorts were seropositive and there was no difference in the SARS‐CoV‐2 total antibodies between the two cohorts. However, cohort 2 had significantly higher levels of ACE2‐blocking Abs and antibodies to the RBD of the WT and VOCs when compared to cohort 1. The high ACE2‐blocking Abs and antibodies to WT in the second cohort could be because they were 6 weeks post second dose compared to cohort 1, which was 12 weeks post second dose. Therefore, it is possible that those in cohort 2 had not entered the contraction phase of the memory response by 6 weeks post second dose and therefore had higher T cell and antibody responses. However, both cohorts were 6 months post first dose, and at that time point, those with a longer dosing gap had higher antibody titers. Although we did not assess neutralizing antibodies in this study, ACE2‐blocking Abs and antibodies to the RBD detected by the HAT assay have been shown to significantly correlate with levels of neutralizing antibodies.^14^, ^21^ Since neutralizing antibodies have been shown to associate with protection against SARS‐CoV‐2 virus infection,^22^ from 6 months post first dose, those with a longer dosing interval appear to have a higher level of protection. An extended dosing gap with the BNT162b2 has also been shown to increase the neutralizing antibody levels with an increase in interleukin‐2 producing CD4 + T cells.^23^, ^24^ Therefore, overall the data appear to suggest that a longer dosing interval may associate with a higher magnitude of neutralizing antibody responses.

We previously showed that the levels of ACE2‐blocking Abs declined from 4 to 12 weeks after a single dose and thereby possibly increasing the susceptibility to infection by 16 weeks.^5^ However, 73.9% still had detectable ACE2‐blocking Abs, while robust T and B cell memory responses were seen in >90% at 16 weeks.^5^ Although there are no data regarding the efficacy of a single dose of the vaccine in preventing infection at ≥16 weeks, we found that 12.5% of this cohort with a longer dosing gap had been infected with the virus, with mildly symptomatic or asymptomatic infection. This is not surprising as Sri Lanka had a high number of cases from April to June due to the alpha variant^25^ and even a higher number of cases with the delta variant from June to the end of September due to the delta variant.^26^, ^27^ Therefore, although a large proportion (11/88) had evidence of infection, the vaccine appeared to have induced an adequate immune response to prevent hospitalization. The waning of neutralizing antibody responses has been observed in especially older individuals with time, and therefore, many countries have recommended a booster dose 6 months after the second dose of the vaccine.^10^, ^28^ Our data show that the increase in the ACE2‐blocking Abs post second dose, compared to 4 weeks post first dose was only significantly higher in the younger age groups (20–39 years old), whereas SARS‐CoV‐2 RBD binding antibodies were significantly higher in older age groups (40–59 years). Therefore, the generation of protective neutralizing antibodies could be age‐dependent, which should be further investigated. However, irrespective of age, those who had a prolonged dosing interval appear to have higher antibody and T cell responses and therefore are likely to have higher neutralizing antibody responses for a longer duration from the time they received the first dose. Therefore, the prolonged gap between the two doses may be beneficial in vaccine roll‐out by enabling more individuals to receive one dose of a vaccine, but not compromising immunity and possibly delaying giving out booster doses.

Interestingly, we found that while only a significant correlation was seen for the total SARS‐CoV‐2‐specific antibodies between the 12 weeks post first dose and 12 weeks post second dose in 20–39 years old and not in cohort 2, the ACE2‐blocking Abs post second dose significantly correlated with the values post first dose. A significant correlation for ex vivo IFNγ ELISpot responses was only observed in cohort 1, where a strong correlation was seen between T cell responses at 12 weeks following the first dose when compared to T cell responses 12 weeks following the second dose. Therefore, individuals who have the highest frequency of responses to the first dose appear to also have a higher magnitude of responses to the second dose. Therefore, the level of immune responses before the second dose appears to be an important factor that determines the magnitude of the responses after the second dose.

Our data show that 97.1% had ACE2‐blocking Abs,12 weeks after post second dose, while 94.2% of those with a 12‐week gap had ACE2‐blocking Abs with median antibody titers of 77.6 (% of inhibition). In contrast, we showed that 12 weeks after the second dose of Sinopharm/BBIBP‐CorV, only 60.9% of individuals had ACE2‐blocking Abs, with median titers of 35.6%.^9^ Therefore, there appear to be significant differences in the kinetics of the immune responses with time, for different types of vaccines. It would be important to take into account these differences, when decisions regarding when and to whom to administer booster doses are taken, to prevent large outbreaks of breakthrough infection. However, although neutralizing antibodies have been shown to associate with protection,^22^ and T cells have been shown to associate with reduced disease severity,^29^ the correlates of a protective immune response are yet unknown. Therefore, the efficacy of different dosing schedules and for different vaccines in providing durable immunity can only be evaluated by clinical trials.

In summary, we have investigated the immune responses following two dosing schedules of the AZD1222 in Sri Lankan individuals. We found that those who had a wider dosing gap had higher antibody and T cell responses, 6 months post first dose of the vaccine when compared to those with a 12‐week dosing gap. It would be important to assess the significance of these differences in immune responses based on the dosing gap on vaccine efficacy.

## Supporting information

Supporting information.
