# Supplementary material for: Kinetics of immune responses to the AZD1222/Covishield vaccine with varying dose intervals in Sri Lankan individuals
Source: Immun Inflamm Dis. 2022 Mar 22;10(4):e592. doi: 10.1002/iid3.592 (PMC8939043; doi:10.1002/iid3.592)
Supplement: Supplementary file 1 — Supporting information. [file IID3-10-e592-s001.docx]

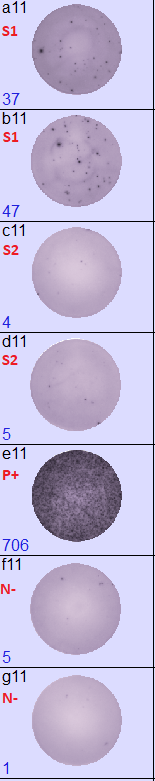


**Supplementary figure 1: An example of an ex vivo ELISpot response at 12 weeks following the second dose of the vaccine.**

S1: 1-130 peptides (representing the 1^st^ half of the spike protein, 130/253)

S2: 131-253 peptides (representing the 2^nd^ half of the spike protein)

P+: positive control

N-: Negative control
